# Core Rehabilitation Outcome Set for Single Sided Deafness (CROSSSD) study: protocol for an international consensus on outcome measures for single sided deafness interventions using a modified Delphi survey

**DOI:** 10.1186/s13063-020-4094-9

**Published:** 2020-03-04

**Authors:** Roulla Katiri, Deborah A. Hall, Nora Buggy, Nicholas Hogan, Adele Horobin, Paul van de Heyning, Jill B. Firszt, Iain A. Bruce, Pádraig T. Kitterick

**Affiliations:** 1 National Institute for Health Research (NIHR) Nottingham Biomedical Research Centre (BRC), Ropewalk House, 113 The Ropewalk, Nottingham, NG1 5DU United Kingdom; 2 Department of Audiology, Mater Misericordiae University Hospital, Dublin, D07 R2WY Ireland; 3 Hearing Sciences, Division of Clinical Neuroscience, School of Medicine, University of Nottingham, Nottingham, NG7 2UH United Kingdom; 4 University of Nottingham Malaysia, Jalan Broga, 43500 Semenyih, Selangor Darul Ehsan Malaysia; 5 Nottingham University Hospitals NHS Trust, Queen’s Medical Centre, Derby Road, Nottingham, NG7 2UH United Kingdom; 6 Department of Otorhinolaryngology, Head and Neck Surgery, Antwerp University Hospital, Antwerp, Belgium; 7 Experimental Laboratory of Translational Neurosciences and Dento-Otolaryngology, Faculty of Medicine and Health Sciences, University of Antwerp, Antwerp, Belgium; 8 School of Medicine, Washington University in St. Louis, St. Louis, Missouri United States of America; 9 Manchester University Hospitals NHS Foundation Trust, Manchester Academic Health Science Centre, Oxford Road, Manchester, M13 9WL United Kingdom; 10 Division of Infection, Immunity and Respiratory Medicine, Faculty of Biology, Medicine and Health University of Manchester, Oxford Road, Manchester, M13 9PL United Kingdom

**Keywords:** Consensus methods, Core outcome set, Delphi technique, Single-sided deafness

## Abstract

**Background:**

Single-sided deafness (SSD) describes the presence of a unilateral severe to profound sensorineural hearing loss. SSD disrupts spatial hearing and understanding speech in background noise. It has functional, psychological and social consequences. Potential options for rehabilitation include hearing aids and auditory implants. Benefits and harms of these interventions are documented inconsistently in the literature, using a variety of outcomes ranging from tests of speech perception to quality of life questionnaires. It is therefore difficult to compare interventions when rehabilitating SSD. The Core Rehabilitation Outcome Set for Single Sided Deafness (CROSSSD) study is an international initiative that aims to develop a minimum set of core outcomes for use in future trials of SSD interventions.

**Methods/design:**

The CROSSSD study adopts an international two-round online modified Delphi survey followed by a stakeholder consensus meeting to identify a patient-centred core outcome domain set for SSD based on what is considered critical and important for assessing whether an intervention for SSD has worked.

**Discussion:**

The resulting core outcome domain set will act as a minimum standard for reporting in future clinical trials and could have further applications in guiding the use of outcome measures in clinical practice. Standardisation will facilitate comparison of research findings.

## Background

‘Single-sided deafness’ (SSD) is the name given to the condition in which there is normal or near-normal hearing in one ear and a severe to profound hearing impairment in the other ear [1]. SSD can be congenital, sudden or progressive. The most common causes of SSD in adulthood are sudden and idiopathic, including vestibular schwannoma [2] and associated surgery [3], Ménière’s disease [4], and sudden-onset sensorineural hearing loss [5]. The incidence of SSD in the United Kingdom is estimated to be approximately 9000 new cases per year [6].

Good hearing in both ears helps people to deal with everyday listening tasks [7]. These include understanding speech in noisy environments and locating where sounds, such as the telephone or car traffic, are coming from [8, 9]. In adults with SSD, both these abilities are compromised [10–14] and can lead to functional [15], psychological and social consequences [16, 17]. The multi-dimensional burden on overall health is indicated by reductions in health-related quality of life [18] in individuals with a diagnosis of SSD.

The most commonly used treatments for SSD restore two-sided (bilateral) access to sounds by re-routing sounds from the impaired ear to the hearing ear [19]. This can be achieved with the help of a specialised hearing aid system known as the CROS (contralateral routing of signals) aid [20]. Bone-anchored hearing aids (BAHA) have also been used as interventions for SSD to achieve signal re-routing [21]. Alternatively, an auditory prosthesis such as a cochlear implant can deliver information about sounds directly to the auditory pathway on the side of the impaired ear, thus creating a sensation of true ‘binaural’ hearing [12].

Existing literature has highlighted inconsistencies in what benefits and risks (side effects) are assessed when evaluating these interventions [22]. The different sorts of benefits and risks are collectively called ‘outcomes’ [23]. For example, researchers have measured aspects or outcomes such as speech understanding in quiet [24–28] or noise [12, 24, 26–45], sound localisation [12, 20, 24, 27–29, 32, 36–41, 43, 44, 46–50], the impact on the recipients’ quality of life [29, 47, 51] or tinnitus effects [12, 31, 52–60]. These inconsistencies in outcomes used in the field of SSD and the variety of methods used to measure them have been identified as a major barrier to synthesising evidence across trials [61]. This diversity in outcomes and instruments used also hinders researchers in making decisions about the choice of outcome measures for health and social care trials of clinical efficacy [23, 62, 63].

The importance of using valid instruments that effectively measure the intended audiological outcomes has been highlighted by Hall et al*.* [64]. Triallists should ideally base the choice of outcome measures on what is important and of interest to people making decisions about healthcare [65–67], not on what outcome instruments are available or most commonly used [68]. If evidence is lacking for an important outcome, this should be acknowledged rather than ignoring the outcome [23]. A core outcome set (COS) developed from the perspectives of healthcare users [69, 70], healthcare professionals and other relevant stakeholders would overcome this problem [71–74].

A COS is defined by COMET (Core Outcome Measures for Effectiveness Trials) as ‘an agreed minimum set of outcomes or outcome measures’ [75]. A COS comprises a standardised collection of outcome domains that should be measured and reported worldwide, at minimum, in all controlled trials within a research area [63, 76–78], as well as a recommended measurement instrument for each outcome domain. An internationally adopted COS allows study findings in a specific health area or condition to be combined, compared and contrasted across trials. It also reduces potential for reporting bias and ensures that the data are useful and usable, which is essential for making well-informed healthcare choices [23].

One of the earliest examples of an attempt to standardise outcomes is an initiative by the World Health Organisation in the 1970s relating to cancer trials [79]. More recent projects are the IMMPACT (Initiative on Methods, Measurement, and Pain Assessment in Clinical Trials) study for chronic pain [80], the OMERACT (Outcome Measures in Rheumatoid Arthritis Clinical Trials) consensus initiative for many rheumatologic conditions [81], the HOME (Harmonizing Outcome Measures for Eczema) framework in dermatology [82], the GASTROS (Standardising Outcome Reporting in Gastric Cancer Surgery Research) study for reporting outcomes in gastric cancer surgery [83], and the COMiT’ID (Core Outcome Measures in Tinnitus International Delphi) initiative for chronic subjective tinnitus [84]. It is vital that all stakeholders, such as healthcare users, with lived experience of the condition, as well as healthcare professionals, commercial representatives or budget holders, are involved in the development of relevant COS [23, 85].

### Aims

The primary aim of the Core Rehabilitation Outcome Set for Single Sided Deafness (CROSSSD) study is to develop an agreed minimum set of outcome domains relevant to both patients and professionals that should be measured and reported in all future trials examining SSD interventions, regardless of whether the intervention restores two-sided (bilateral) access to sound via the better ear or delivers sound information directly to the impaired ear.

The primary objective is as follows:

- To develop an international consensus on a COS for SSD interventions using a long-list list of candidate outcomes, a two-round modified electronic Delphi survey and a subsequent face-to-face consensus meeting with relevant stakeholders

To assist in identifying potential measurement instruments for each core outcome domain identified in the first objective, two secondary objectives are as follows:

- To synthesise the evidence on the available outcome measurement instruments for measuring the construct outcomes (e.g., speech perception, localisation) in the COS for SSD interventions
- To follow up on any issues raised during the Delphi process concerning the concept definition of any of the outcome domains in a subsequent face-to-face workshop involving healthcare users and healthcare professionals

## Methods/design

This study will adopt recommendations by the COMET Initiative [63, 72, 86–88] and the COMET Handbook version 1.0 [23] '(see Additional file 2 COS-STAP checklist). We will use a modified e-Delphi process to achieve a consensus of opinion among broadly representative and international expert stakeholder groups [89]. This study uses an observational design and is sponsored by the University of Nottingham and managed by the National Institute for Health Research (NIHR) Nottingham Biomedical Research Centre (BRC). A prospective study protocol was registered on the COMET database in January 2018 [90]. This paper describes protocol version 2.0 (dated 6th July 2019) that was approved by the Proportionate Review Nottingham 2 Research Ethics Committee (REC reference 19/EM/0222, IRAS Project ID 239750) on the 6th of August 2019.

### Research steering group

A research steering group was appointed in October 2017 to guide the protocol development and oversee the CROSSSD study. The group comprises international colleagues who are experts in SSD research methodologies and intervention approaches (PVH, JBF, IAB); a patient and public involvement (PPI) and engagement manager (AH); two healthcare users with lived experience of SSD, referred to as public research partners (NB, NH); and the study management team (RK, DAH, PTK).

The roles of the research steering group are as follows:

1. Support the development of the study protocol, specifically commenting on the feasibility of the modified Delphi process, reviewing study documentation (e.g., advertisements, information leaflets, supporting video explanations of the survey and intended advertisements, website content) and participating in a pilot of round 1 of the e-Delphi survey
2. Review the initial list of outcome domains and associated descriptions, specifically commenting on the readability of the outcome descriptions, the appropriateness of the grouping of outcomes into categories and providing any additional outcomes that they believe should be included in round 1 of the e-Delphi survey
3. Assist with participant recruitment and engage in dissemination activities, such as contributing to publications
4. Consider any necessary revisions to the protocol which may inadvertently arise while the study is underway

### Eligibility criteria for Delphi panels

A range of expertise within the panel is an important quality criterion for development of a core outcome domain set [23]. Specific inclusion criteria have been defined for three key stakeholder groups:

1. *Healthcare users* who have experience of living with SSD for 12 months or more and have received or have considered receiving an SSD intervention
2. *Healthcare practitioners* who have a clinical qualification, are currently employed by a public or private institution that provides SSD interventions to patients and have experience of assessing, diagnosing or managing SSD in adults
3. *Clinical researchers* who have an academic qualification, are currently employed by a research organisation, have current or ‘recent past’ experience with studies that focus on questions of clinical efficacy (benefit) of SSD interventions in humans (i.e., co-author on a relevant peer-reviewed journal publication in the past 3 years)

Other participants will be invited to participate, including those commercial representatives who are currently employed by a company that develops, manufactures or sells product(s) that may be used as an SSD intervention, as well as funders who are currently employed by an organisation that funds SSD research and have experience of reviewing funding applications for SSD intervention research in the last 3 years. However, we do not anticipate recruiting sufficient numbers from among these stakeholders to form distinct stakeholder groups in their own right, because these pods of stakeholders are small.

General eligibility for participation includes men and women aged 18 years or older who are computer-literate; possess sufficient command of English to read, understand and independently complete the questionnaires; and have the ability to give informed consent. All enrolled e-Delphi panellists will be eligible to register their interest in attending a 1-day face-to-face consensus meeting and/or a follow-up workshop. However, allocation of places will be limited to those respondents who complete both rounds of the e-Delphi survey. None of the research steering group members will be allowed to vote on domains in the consensus meeting, because this risks inadvertently introducing a power differential across participants; however, they can enrol in the e-Delphi surveys.

### Panel size and justification

There is no agreed method to statistically calculate a sample size for e-Delphi surveys or consensus meetings [85]. However, one of the key deciding factors is that the participant panel membership should adequately represent corresponding stakeholder groups. Adult SSD is a relatively rare hearing disorder, with approximately 9000 new cases diagnosed in the United Kingdom each year [6]. SSD intervention is also a relatively new field, especially cochlear implantation, which has been used in this population only in the last decade [91]. Therefore, the number of professionals and members of the public with knowledge and experience of these interventions is limited. The aim is therefore to recruit a sufficient number of participants so that a minimum of 20 participants complete the two rounds of the e-Delphi survey in each of the key stakeholder groups (healthcare users, healthcare practitioners and clinical researchers). This target is consistent with our previous work [85].

The consensus meeting and follow-up workshop require in-depth discussions, and therefore up to 20 participants will be recruited for each. Enrolment will be balanced across stakeholder groups when possible.

### Recruitment methods

Effective recruitment methods similar to the ones described by Hall et al. in 2018 [85] will be used. For example, adopting an explicit marketing plan and engaging with charities or participants to act as ‘champions’ were successful strategies that helped recruitment for both healthcare users and professionals in the COMiT’ID study.

Generally, the CROSSSD study recruitment plan includes e-promotion routes, which include a study webpage (www.nottingham.ac.uk/research/groups/hearingsciences/projects/crosssd/index.aspx) and regular updates on the study’s progress via social media platforms (e.g., Twitter @CROSSSD_, @hearingnihr). A video advertisement promoting the study will also be developed.

Healthcare users will be targeted using various routes, including promotion of the study during the Ménière’s Society Balance Awareness Week (September 2019) and the British Acoustic Neuroma Association annual conference (October 2019). Moreover, healthcare users in the United Kingdom and Ireland will be targeted using a traditional National Health Service (NHS) recruitment route with 18 audiology and ear, nose and throat (ENT) departments whose members specialise in provision of interventions for SSD. These will be designated as participant identification centres (PICs) and will be in addition to the lead site in Nottingham. An application will also be submitted for adoption of the CROSSSD study into the NIHR Clinical Research Network (CRN) portfolio, through which other NHS sites can express their interest to support the study by being a PIC.

PICs will display study posters in the audiology and ENT clinic waiting rooms and hand out participant information leaflets, as appropriate. If feasible, participant invitation letters will be posted by local PIC clinicians to their database of patients diagnosed with SSD. Specific e-promotion routes include several organisations that have agreed to support the project by publishing newsletter articles and announcements to their members (e.g., Manchester Hearing BRC volunteers, Ménière’s Society). Finally, the lead study site, the NIHR Nottingham BRC, has a participant database containing email contacts for approximately 70 healthcare users who have been diagnosed with SSD and will be invited to participate.

For healthcare professional recruitment, we are aiming to recruit experts who maximise the international relevance of the study findings [89]. A number of professional networks and organisations will be approached to circulate invitations to their membership (e.g., British Society of Audiology Adult Rehabilitation Interest Group, HEARRING Network, Hearing Australia). When possible, the expert members of the CROSSSD steering group will be asked to approach their networks to make the approach more personal. Parallel routes for recruiting healthcare professionals will also involve personal invitation via email or face-to-face contact (e.g., presentations at teams’ monthly journal clubs or research meetings).

The study management team has created a long list of potential participants with relevant expertise via existing connections with the NIHR Nottingham BRC, manual searches of relevant hearing-related organisations (e.g., UHealth Ear Institute at the University of Miami), corresponding authors of the relevant research publications identified by the systematic review [90], manual searches of relevant conference proceedings in the last 3 years (e.g., UK Implantable Acoustic Devices Conference, International Conference on Cochlear Implants and Other Implantable Technologies [Ci2018.org], OSSEO International Congress on Bone Conduction Hearing and Related Technologies), and email queries sent to representatives from each additional stakeholder organisations from commercial sectors (e.g., clinical research managers for relevant device companies) and funding bodies asking for recipients to nominate any colleagues with expertise in SSD interventions.

Eligible professionals will be identified and invited to participate and will be asked a number of questions which will confirm both their stakeholder group (i.e., job role or medical specialty) and that they meet the eligibility criteria for their stakeholder group (*see* ‘Eligibility criteria for Delphi panels’ section above). Professional stakeholder groups include individuals involved in management or research in the field of SSD. These include healthcare professionals, clinical researchers, commercial representatives, funders, and journal editors. These groups have been identified as those representing the main professional categories in SSD research and clinical trials.

### Delphi survey

**Fig. 1:**
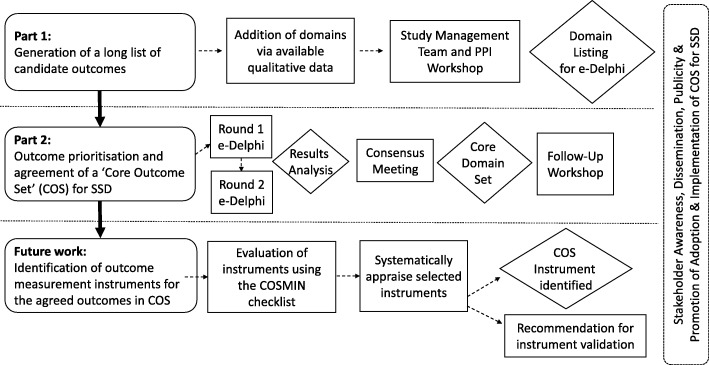
CROSSSD study plans for the development of an internationally agreed core outcome set (COS) for single-sided deafness interventions, identification of outcome measurement instruments, and adoption and implementation of the COS

An international Delphi survey will be managed online using DelphiManager software maintained by the COMET Initiative [92]. Each panellist will receive a unique identification code and an e-link to the webpage. A video explanation will illustrate how to use the online tool. A flowchart of the study is shown in Fig. 1.

Delivering this study online allows us to capture the opinions of a diverse population of stakeholders with an interest in shaping outcome measures for SSD interventions. If any healthcare users do not have home access to a computer or a tablet, then they will be offered the option of visiting the NIHR Nottingham BRC to complete the two online rounds using one of the centre’s computers.

The e-Delphi technique can minimise response bias because individual feedback is anonymised and not affected by views of influential individuals [93]. Surveys can also be perceived as intimidating by members of the public as a result of the long number of outcomes included in some e-Delphi surveys that lay participants would have to go through and score at every round [94]. Methodological features highlighted by Smith et al*.* [95], such as shortening and renaming the long list of domains and plain language descriptions, will be adhered to when possible. Evaluations discussed by Hall et al. [85] will be considered to ensure robust recruitment and retention of healthcare users.

### Part 1: Preparatory work to generate the long list of candidate outcomes

Potentially important outcomes were first gathered from a systematic review of the literature which identified those outcome domains and outcome instruments reported in studies investigating interventions that seek to restore hearing in adults with SSD [96], as well as by considering published qualitative data [16] derived from group interviews examining subjective psychological and social effects of highly asymmetric hearing loss. A workshop with members of the research steering group reviewed this long list of candidate outcomes with the following objectives:

1. Exclude outcomes that are deemed outside the scope of this COS
2. Identify any missing outcomes
3. Consider the choice of language used to define each outcome
4. Generate plain-language descriptions of each outcome

The CROSSSD study management team collated all primary and secondary outcomes that were identified by the systematic review [96], which equated to 216 outcome domain terms. In preparation for the workshop, these were categorised into six preliminary groupings: (1) adverse effects or harms, (2) performance in a test situation, (3) patient outcome, (4) resource use, (5) satisfaction and (6) other/cannot code. All individual outcome domains were printed on cards in preparation for a 2-day workshop that took place in July 2019 with members of the research steering group (RK, DAH, AH, PTK) and the two public research partners (NH, NB).

During the workshop, each member first performed an independent rapid review of the individual outcome domains and marked those that they thought did not fit within the scope of core domain set for SSD interventions. It was agreed that an outcome domain would be excluded for not fitting within the scope if all six members unanimously agreed to exclude or there was no more than one dissenting opinion. During this rapid review, 83 outcome domain terms were excluded, and examples of those domains were phrases describing ‘how’ to measure such as *thresholds, audiologic, tonotopy, informational masking, cortical changes*, and *brain activity*; or outcome domains that were deemed too broad, generic or ill-defined, such as *qualities, hearing, therapy, background noise, mental health,* and *cognitive distress*. Further group discussion led to consolidation of an additional 23 outcome domain terms into a smaller number of outcome domain labels. For example, *hearing disability* was considered to be synonymous with *residual disability, perceived hearing disability, hearing disability at everyday life,* and *auditory disability*. Another 17 outcome domain terms were deemed to be duplicates or descriptions of already-included outcome domains (e.g., *subjective assessment of handicap, disability, use, benefit*, and *satisfaction*). This left 93 outcome domains for the long list.

**Fig. 2:**
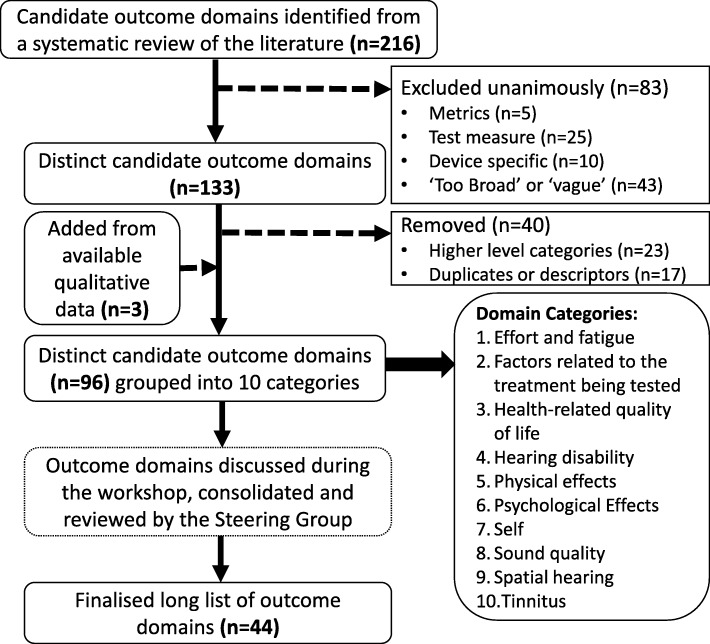
CROSSSD study flow diagram illustrating the pre-Delphi stage that has been completed during a workshop with members of the research steering group

The workshop team next systematically reviewed and discussed the findings published by Lucas et al*.* [16] to determine whether qualitative interviews might have identified any other candidate outcome domains. This process added three new outcome domains which had not been assessed explicitly in previous quantitative studies: *personal safety* (e.g., road safety, independent living), *motivation* (e.g., to engage in challenging listening situations) and *mood* (e.g., general sense of well-being). The resulting 96 outcome domains were consolidated further by grouping domains together that were considered by the group to describe the same domain. For example, the outcome domains *sound localisation, localisation, localisation performance, azimuthal sound localisation, auditory localisation, localisation ability, source localisation, localisation testing,* and *ability to judge direction of sound* were consolidated into an outcome domain labelled ‘*sound localisation* (telling where a sound is coming from)’. This consolidation resulted in a final list of 43 outcome domains, which were subsequently organised thematically into ten categories: (1) *psychological effects*, (2) *factors related to the treatment being tested*, (3) *health-related quality of life*, (4) *hearing disability*, (5) *spatial hearing*, (6) *physical effects*, (7) *self*, (8) *sound quality*, (9) *tinnitus* and (10) *other effects* (Fig. 2).

Some of the outcome domains were the same as had been defined in our previous work on tinnitus [84] and hearing loss [97], so we used the same plain-language descriptors when appropriate. Others required plain-language descriptors to be developed through interactive discussion during the workshop.

The long list of outcome domains, labels and plain-language descriptors were subsequently circulated electronically to the CROSSSD study steering group for feedback and cross-checking. This was done to ensure that the outcome domain concepts were explained in ways that are understandable and meaningful to an international audience, especially to those whose first language is not English. The research steering group was also prompted to suggest any missing candidate outcome domains. The study management group used this feedback and, following further revisions to the long list, prepared a final list of 44 candidate outcome domains that will be incorporated into the e-Delphi survey (see Additional file 1 for the final list of outcome domains and their definitions). The randomisation feature of the DelphiManager software (version 4.0) will be used to avoid potential weighting [98]; that is, presentation of each outcome domain will be randomised as per current recommendations [23, 99].

### Part 2: Outcome prioritisation and consensus decision-making

The modified e-Delphi survey comprises a series of two sequential questionnaires or ‘rounds’ aiming to obtain a consensus of opinion from professional and healthcare user stakeholder groups. Each Delphi survey will be managed using a bespoke online e-management system (DelphiManager software, version 4.0) maintained by the COMET Initiative [92]. Both survey rounds will contain a questionnaire that includes the final long list of categorised outcome domains (*n* = 44) developed in part 1. International healthcare users and professionals with experience in receiving or managing SSD interventions will be identified and invited to take part (*see* ‘Recruitment methods’ section above for details).

Upon entering the online survey webpage, an introductory page will reiterate key information previously provided in the participant information sheet, including an embedded link to a video explanation. Participants will then be asked to give informed consent, and a unique identification code will be generated to allow tracking of individual responses in round 2. Video explanations will guide participants through the round. Following this, participants will complete a checklist of relevant personal characteristics. These include personal and/or professional experience with SSD interventions; treatments trialled, if applicable (e.g., BAHA, CROS); the group they primarily identify with (e.g., healthcare users, healthcare practitioners, clinical researchers, commercial representatives, funders); age range; gender; country of residence; primary language used for communication; professional role (if applicable); length of SSD diagnosis and interventions primarily used (for healthcare users); and email address.

The survey will be piloted by the study management team and public research partners for face validity, understanding and acceptability. Following this, if needed, modifications will be made before finalising and launching the questionnaire. When the first round of the e-Delphi survey is launched, participant recruitment will commence immediately, and the recruitment period will be for at least 2 months. Participant response rates will be monitored throughout, and the study management team will keep clearly defined records of the number of participants who have completed the rounds and those who have not.

### Round 1

For each of the 44 outcome domains, participants will be asked to think about the importance of each and indicate how important it is to measure when deciding if an intervention is working. Participants will be asked to assign a score to each of the 44 candidate outcome domains. A 9-point Likert scoring system will be used, with a score of 1 to 3 signifying that an outcome domain is of *limited importance*, 4 to 6 indicating *important but not critical*, and 7 to 9 meaning *critical and important* [100]. Participants will be made aware that an outcome domain will be considered for inclusion in the COS only if 70% or more of the participants in each of the stakeholder groups select scores 7–9 on the scale. If a participant feels that (s)he did not understand a particular outcome, (s)he will be able to select ‘*unable to score*’.

Following each outcome and at the end of the questionnaire, each participant will be offered an open-text box to add any comments about particular outcome domains. This is optional, but participants will be encouraged to provide a reason for their scores on individual outcomes as recommended by the COMET Handbook [23]. These comments will be summarised as part of the feedback after the first round.

In round 1, participants will be able to propose additional outcome domains. These additional outcome domains will be reviewed and coded by the study management team members, with appropriate plain-language concept definitions, to ensure that they represent new items for inclusion in round 2. When uncertainty exists, the research steering group will be consulted, and all new outcome domain terms, concept definitions and category labels will be reviewed. Adhering to current recommendations, reporting of the e-Delphi surveys will describe any new outcomes introduced into the consensus process at the end of round 1, with reasons [101].

### Round 2

Participants will be eligible to continue to round 2 if they have scored at least 22 (50%) of the outcome domains in round 1. Corresponding data from those participants who responded to fewer than this will be removed. In round 2, all participants will receive the same list of outcomes with feedback tailored according to their key group allocation (healthcare users, healthcare professionals, clinical researchers). Participants who identify themselves as commercial representatives or funders when they register will be considered collectively, and feedback on their scores will be reported separately from the three stakeholder groups.

The purpose of round 2 is to enable participants to reflect on their scores in light of the viewpoint of their stakeholder group and the other stakeholder groups in the e-Delphi survey. Results will be presented graphically as well as numerically to be readily understood by participants. Participants will be asked to re-score the same list of outcome domains, considering this new information. To help give meaning to the 9-point Likert scale [100], participants will be reminded that individual outcome domains will be considered for inclusion in the COS only if 70% of all participants select scores of 7–9 on the scale. The distribution of the new scores for each outcome domain will then be calculated for each stakeholder group. Other aspects of design and analysis are the same as for round 1. After completion of the second round of the e-Delphi survey, a questionnaire, anticipated to take up to 10 min to complete, will be emailed to all participants to collect feedback on their experience of being a participant.

### Consensus meeting

The aim of the consensus meeting is to integrate healthcare users and professional perspectives on outcomes, as well as to provide final recommendations on an agreed COS for SSD interventions. Participants who have completed the two rounds of the e-Delphi survey, responded to at least 90% of the outcome domains in round 2, and register an interest in participating in the consensus meeting will be eligible to participate. Places will be allocated on a first-come, first-served basis. Recruitment will be guided by methods successfully adopted by Fackrell et al*.* [102]; that is, as far as possible, allocated places will maintain a 50/50 balance across healthcare users and professionals and will aim to include non-UK, non–native English language speakers. As far as possible, the COMET guidance for designing an accessible COS consensus meeting will be followed [103].

After confirming their attendance to the meeting, participants will be sent an email with information on how to get to the meeting and what to expect, as well as the participant information sheet again as a reminder. At the consensus meeting, the research team will discuss with the participants the aim of the meeting and what will happen, ensuring that all participants understand the purpose before consenting and starting the meeting.

An experienced independent moderator will be recruited to facilitate the consensus meeting discussions to agree to a final COS. Discussion within the meeting will include anonymised voting on each outcome as either ‘in’ or ‘out’ (e.g., using electronic keypads which will create histograms and descriptive statistics ‘live’, to be displayed in the meeting). Participants will be given materials summarising the anonymised round 2 results.

### Consensus criteria

Consensus recommendations will be guided by round 2 results. The ‘70/15%’ consensus approach as described by Williamson et al*.* [71], and Williamson et al*.* [23] and successfully used by Harman et al*.* [104] and Hall et al*.* [84] will be employed, as follows:

- For outcomes recommended to be included on the basis of round 2 analysis (70% scored 7–9), the moderator will establish whether anyone has a major reason to want any to be excluded. The moderator will focus the discussion and voting on these outcomes. Domains will be included if at least 70% of participants vote ‘in’. All other outcomes recommended for inclusion will be ‘in’, without further discussion.
- For outcomes in which at least 50% of more than one stakeholder group scored 7–9 on the round 2 analysis, the moderator will focus the discussion and voting. Domains will be included if at least 70% of participants vote ‘in’.
- For outcomes in which less than 50% of the participants in all stakeholder groups scored 7–9 on the round 2 analysis, the moderator will establish whether anyone has a major reason to want any to be included. Domains will be included only if at least 70% of participants vote ‘in’.

If consensus is not reached after two rounds of voting, a ‘majority rules’ approach will be applied. Because time for discussion will be limited, there will be no discussion about outcomes whereby the round 2 data meet the criteria for exclusion based on the pre-defined consensus definition.

The final consensus meeting will be audio recorded and transcribed to facilitate reporting. These will be classed as source data and will be retained in the study archives using unique identifier codes for each talker. Reporting of the Delphi surveys will list the outcomes in the final COS [101].

Finally, we will evaluate the participants’ experience of the consensus meeting using a short evaluation form. This is adapted and modified from the recommended template developed by the COMET Initiative [105]. It is anticipated that completion of this will take approximately 10 min, and completion is entirely voluntary.

### Follow-up workshop

This workshop will be convened only if considered necessary by the study management team, a judgement that will be made in consultation with the research steering group. The aim of the workshop would be to discuss in more detail any of the outcome domains that were voted into the core outcome domain set but for which there might have been some unresolved debate about what the exact concept of the domain was or how it was defined in the plain-language descriptor. This will be important underpinning information to have before seeking to identify suitable measurement instruments for each of the outcome domains in the COS.

### Analysis

Compliance in the e-Delphi survey will be defined according to the number of participants completing rounds 1 and 2. Participation within each stakeholder group will be assessed, including (1) numbers who were directly contacted, (2) numbers who registered in the e-Delphi system, (3) numbers enrolled, and (4) numbers completing each round. Similar to the methods used by the mOMEnt (management of Otitis Media with Effusion in children with cleft palate) team [106], if a reduced number of responders (*n* < 10) is observed for one or more stakeholder groups, the round 2 Delphi survey will be reviewed and revised. For example, we may consider amalgamating stakeholder groups.

Other analysis will incorporate participant characteristics, such as gender, country, region and native English language speaker (or not). We will analyse the shifts in scores between rounds 1 and 2 for each outcome domain and stakeholder group as a consequence of considering the anonymised feedback from other participants.

Attrition, referring to the percentage of participants who withdraw or drop out between rounds, will be analysed and reported using methods similar to those employed by the mOMEnt team [106] and the COMiT’ID team [84]. For example, attrition bias which might occur if participants who do not respond in round 2 have different views from their stakeholder group peers who participate in both rounds [23] will be considered and analysed. To achieve this, methods used by Bruce et al*.* [107] can be adopted: Response distributions of withdrawn and completing participants can be drawn. Graphical representations by stakeholder group (healthcare users, healthcare professionals, clinical researchers) can be drawn, too, as presented by Hall et al*.* [108], to indicate if attrition bias is likely to have affected the outcome domain recommendations.

Round 2 score distributions for each outcome domain will be considered at the final consensus meeting using a nominal group technique to evaluate individual perspectives. For example, like the methods adopted by Harman et al*.* [106], the results of the stakeholder group responses will be compared with the whole group’s response, and percentage agreement will be considered to plan the focus of the consensus meeting [84]. The data derived from the Delphi feedback questionnaire and consensus meeting evaluation form will comprise open-text responses, and these will be analysed using a thematic analytic approach.

### Dissemination

The project proposal is registered in the COMET Initiative database [90]. Data derived from the final analysis of the e-Delphi survey, consensus meeting and follow-up workshop will be presented at relevant national and international conferences such as the British Society of Audiology e-conference and the Implantable Acoustic Devices Conference in Oxford (September 2020). Peer-reviewed publications resulting from the research are also planned. We intend to publish the final COS addressing all primary objectives in the summer of 2020. This research will be further disseminated to members of the public and clinicians through specialist magazine articles and support groups. Participants will not be identified in any publications.

## Discussion

This paper describes the design of a Delphi process to develop a COS for SSD interventions, comprising an agreed minimum set of outcome domains relevant to both patients and professionals. This will be applicable to all future trials examining SSD interventions, regardless of whether the intervention restores two-sided (bilateral) access to sound via the better ear or delivers sound information directly to the impaired ear.

During the study protocol development, a question arose whether to consider developing a single COS for the two intervention approaches (re-routing/restoring) or whether to develop separate COSs, one for each intervention approach. The CROSSSD study research steering group was asked to consider the advantages and disadvantages and to help make a decision.

Advantages of considering both intervention approaches together were as follows:

- A single COS would set a standard for outcomes that are critical and important for any of the common intervention strategies. This would facilitate comparisons across intervention methods.
- A single minimum reporting standard may encourage uptake, minimise the cost and time resources required, and reflect the fact that SSD is a relatively small field in otology with a limited number of clinical trials whose designs and methodological quality are highly variable [64].
- A single Delphi survey improves confidence with regard to adequate numbers of stakeholders recruited internationally to ensure that the decision-making represents a wider view.

Disadvantages of considering both intervention approaches together were as follows:

- Outcomes common to both intervention approaches might be less specific to the unique benefits of either intervention. The chosen outcomes might not be optimally sensitive to detecting treatment-related change.
- Seeking a single COS might reduce the potential for reaching consensus criteria on individual outcome domains because the different intervention approaches can be very different in their intended effects.
- Few participants are likely to have expertise in both intervention approaches.
- Two separate Delphi surveys would potentially deliver a stronger message because they would be tailored to individual approaches: re-routing and restoring interventions.

Following thorough consideration of the arguments put forward, it was agreed to proceed with a single consensus process to develop one COS that would be applicable to both intervention approaches. Similar issues have previously been considered by the IMMPACT (Initiative on Methods, Measurement, and Pain Assessment in Clinical Trials) group [109] and led to the same decision. The IMMPACT team proposed that development of a single core set of domains and measurement procedures would facilitate the comparison and pooling of data while leaving investigators free to augment the core domains with other outcomes of their choice.

To our knowledge, this is the first time that the Delphi technique using these consensus decision-making methods has been used for developing a COS in SSD. This study also heavily incorporates patient and public involvement throughout all stages of the project. An agreement on a set of outcome domains for what is critical and important for deciding whether an intervention is efficacious will drive up the quality and relevance of research by ensuring that the most relevant outcomes are consistently measured and reported in every clinical trial relating to SSD. This would make it much easier for people with SSD and their intervening clinicians to make sense of all the knowledge produced and consequently minimise bias when making decisions about healthcare. This should subsequently lead to improvements in SSD interventions and in turn the management and clinical outcomes of patients with SSD. On the basis of the recommended outcome domains, further research will then be needed to identify measurement instruments that assess the outcome domains in the minimum set. 

## Supplementary information

**Additional file 1.** Table of domain categories, SSD intervention-related outcome domains, and concept definitions for all 44 outcome domains co-produced with collaborators with lived experience of SSD at the pre-Delphi stage.

**Additional file 2.** Core Outcome Set-STAndardised Protocol Items (COS-STAP) Checklist.

## Data Availability

Upon the completion of the study, supporting data will be available upon request.

## Abbreviations

BAHA: Bone-anchored hearing aids
BRC: Biomedical Research Centre
COMET: Core Outcome Measures for Effectiveness Trials
COMiT’ID: Core Outcome Measures in Tinnitus, International Delphi
COS: Core outcome set
CRN: Clinical Research Network
CROS: Contralateral routing of signals
CROSSSD: Core Rehabilitation Outcome Set for Single Sided Deafness study
COS-STAP: Core Outcome Set-STAndardised Protocol
ENT: Ear, nose, and throat
GASTROS: Standardising Outcome Reporting in Gastric Cancer Surgery Research
HOME: Harmonising Outcome Measures for Eczema
IMMPACT: Initiative on Methods, Measurement, and Pain Assessment in Clinical Trials
mOMEnt: Management of Otitis Media with Effusion in children with cleft palate
NHS: National Health Service
NIHR: National Institute for Health Research
OMERACT: Outcome Measures in Rheumatoid Arthritis Clinical Trials
PIC: Participant identification centre
PPI: Patient and public involvement
SSD: Single-sided deafness
