# Correction to: Core Rehabilitation Outcome Set for Single Sided Deafness (CROSSSD) study: protocol for an international consensus on outcome measures for single sided deafness interventions using a modified Delphi survey

**DOI:** 10.1186/s13063-020-04240-2

**Published:** 2020-03-17

**Authors:** Roulla Katiri, Deborah A. Hall, Nora Buggy, Nicholas Hogan, Adele Horobin, Paul van de Heyning, Jill B. Firszt, Iain A. Bruce, Pádraig T. Kitterick

**Affiliations:** 1 National Institute for Health Research (NIHR) Nottingham Biomedical Research Centre (BRC), Ropewalk House, 113 The Ropewalk, Nottingham, NG1 5DU UK; 2 Department of Audiology, Mater Misericordiae University Hospital, Dublin, D07 R2WY Ireland; 3 Hearing Sciences, Division of Clinical Neuroscience, School of Medicine, University of Nottingham, Nottingham, NG7 2UH UK; 4 University of Nottingham Malaysia, Jalan Broga, 43500 Semenyih, Selangor Darul Ehsan Malaysia; 5 Nottingham University Hospitals NHS Trust, Queen’s Medical Centre, Derby Road, Nottingham, NG7 2UH UK; 6 Department of Otorhinolaryngology, Head and Neck Surgery, Antwerp University Hospital, Antwerp, Belgium; 7 Experimental Laboratory of Translational Neurosciences and Dento-Otolaryngology, Faculty of Medicine and Health Sciences, University of Antwerp, Antwerp, Belgium; 8 School of Medicine, Washington University in St. Louis, St. Louis, Missouri USA; 9 Manchester University Hospitals NHS Foundation Trust, Manchester Academic Health Science Centre, Oxford Road, Manchester, M13 9WL UK; 10 Division of Infection, Immunity and Respiratory Medicine, Faculty of Biology, Medicine and Health University of Manchester, Oxford Road, Manchester, M13 9PL UK

**Correction to: Trials**

**https://doi.org/10.1186/s13063-020-4094-9**

Following the publication of our article [1], the authors have notified us of a typo in the third bullet point of the Consensus Criteria section.

- Original phrase:

“For outcomes in which less than 50% of the participants in all stakeholder groups scored **1–7** on the round 2 analysis (…)”

- Corrected phrase:

“For outcomes in which less than 50% of the participants in all stakeholder groups scored **7–9** on the round 2 analysis (…)”

Also, the link included in the Funding section, right after “the 15th International Conference on Cochlear Implants and Other Implantable Auditory Technology” was removed.

The original article has been corrected.
