# Supplementary material for: Core Rehabilitation Outcome Set for Single Sided Deafness (CROSSSD) study: protocol for an international consensus on outcome measures for single sided deafness interventions using a modified Delphi survey
Source: Trials. 2020 Mar 4;21:238. doi: 10.1186/s13063-020-4094-9 (PMC7057560; doi:10.1186/s13063-020-4094-9)
Supplement: Supplementary file 2 — Additional file 2. Core Outcome Set-STAndardised Protocol Items (COS-STAP) Checklist. [file 13063_2020_4094_MOESM2_ESM.docx]

**COS-STAP Checklist**

| **Checklist Item** | **Item No** | **Checklist Item Details** | **Where item can be found in submitted manuscript** |
| --- | --- | --- | --- |
| TITLE / ABSTRACT | | | |
| Title | **1a** | Identify in the title that the paper describes the protocol for the planned development of a COS | Lines 4-6 |
| Abstract | **1b** | Provide a structured abstract | Page 2 |
| INTRODUCTION | | | |
| Background and objectives | **2a** | Describe the background and explain the rationale for developing the COS, and identify the reasons why a COS is needed and the potential barriers to its implementation | Pages 3-5 |
|  | **2b** | Describe the specific objectives with reference to developing a COS | Lines 125-136 |
| Scope | **3a** | Describe the health condition(s) and population(s) that will be covered by the COS | Lines 65-69 |
|  | **3b** | Describe the intervention(s) that will be covered by the COS | Lines 77-82 |
|  | **3c** | Describe the context of use for which the COS is to be applied | Lines 92-99 |
| METHODS | | | |
| Stakeholders | **4** | Describe the stakeholder groups to be involved in the COS development process, the nature of and rationale for their involvement and also how the individuals will be identified; this should cover involvement both as members of the research team and as participants in the study | Lines 174-198 |
| Information sources | **5a** | Describe the information sources that will be used to identify the list of outcomes. Outline the methods or reference other protocols/papers | Pages 11-15 |
|  | **5b** | Describe how outcomes may be dropped/combined, with reasons | Pages 11-15 |
| Consensus process | **6** | Describe the plans for how the consensus process will be undertaken | Pages 14-16 |
| Consensus definition | **7a** | Describe the consensus definition | Pages 16-18 |
|  | **7b** | Describe the procedure for determining how outcomes will be added/combined/dropped from consideration during the consensus process | Pages 17-18 |
| ANALYSIS | | | |
| Outcome scoring / feedback | **8** | Describe how outcomes will be scored and summarised, describe how participants will receive feedback during the consensus process | Pages 18-19 |
| Missing data | **9** | Describe how missing data will be handled during the consensus process | Page 19 |
| ETHICS and DISSEMINATION | | | |
| Ethics approval / informed consent | **10** | Describe any plans for obtaining research ethics committee/institutional review board approval in relation to the consensus process and describe how informed consent will be obtained (if relevant) | Lines 563-567 |
| Dissemination | **11** | Describe any plans to communicate the results to study participants and COS users, inclusive of methods and timing of dissemination | Lines 503-510 |
| ADMINISTRATIVE INFORMATION | | | |
| Funders | **12** | Describe sources of funding, role of funders | Lines 579-586 |
| Conflicts of interest | **13** | Describe any potential conflicts of interest within the study team and how they will be managed | Lines 575-577 |
